# CAM-net: a context-aware network for identifying reliable microbial relations via propagation cliques

**DOI:** 10.1093/bib/bbaf631.041

**Published:** 2025-12-12

**Authors:** Junhui Zhang, Wenfei Xu, Jieqi Xing, Yangyang Sun, Shi Huang, Xiaoquan Su

**Affiliations:** College of Computer Science and Technology, Qingdao University, Qingdao, China; College of Computer Science and Technology, Qingdao University, Qingdao, China; College of Computer Science and Technology, Qingdao University, Qingdao, China; College of Computer Science and Technology, Qingdao University, Qingdao, China; Faculty of Dentistry, The University of Hong Kong, Hong Kong SAR, China; College of Computer Science and Technology, Qingdao University, Qingdao, China

## Abstract

**Motivation:**

The human gut microbiome is a complex ecosystem essential to host health, yet its interspecies interactions remain incompletely understood. Conventional correlation analyses typically rely on isolated pairwise measures (e.g., Spearman correlation or SparCC), which overlook the broader ecological context. This narrow perspective often produces spurious associations in large-scale datasets, obscuring genuine microbial interactions.

**Methods:**

Here we present CAM-Net, a context-aware microbial correlation framework that fundamentally departs from pairwise approaches. Instead of treating associations in isolation, CAM-Net dynamically constructs a ‘local environment’ around a target microbe and infers relational propagation cliques that reflect ecologically meaningful interactions. By explicitly controlling for community background, CAM-Net reduces false positives and captures context-dependent ecological dynamics such as facilitation and competition.

**Results:**

We applied CAM-Net to whole-genome sequencing (WGS) and 16S rRNA gene sequencing datasets encompassing over 30,000 human gut microbiomes, focusing on two microbes: *Akkermansia muciniphila* and *Lactobacillus acidophilus*. For *A. muciniphila*, an autochthonous bacterium, CAM-Net identified a relational propagation clique that was reproducible across independent datasets, underscoring its symbiotic ecological role. In contrast, for *L. acidophilus*, an exogenous species of the human gut, CAM-Net recovered only a weakly correlated clique, consistent with prior knowledge of its transient colonization. Notably, neither species produced stable associations in 16S datasets, reflecting limitations of amplicon sequencing—including data heterogeneity, batch effects, and restricted taxonomic resolution.

**Conclusion:**

These findings show CAM-Net as a robust, context-aware alternative to conventional correlations, moving beyond sparse pairwise comparisons to infer microbial interactions and decode microbial ecology.

